# Interfacing the Core-Shell or the Drude Polarizable Force Field With Car–Parrinello Molecular Dynamics for QM/MM Simulations

**DOI:** 10.3389/fchem.2018.00275

**Published:** 2018-07-10

**Authors:** Sudhir K. Sahoo, Nisanth N. Nair

**Affiliations:** Department of Chemistry, Indian Institute of Technology Kanpur, Kanpur, India

**Keywords:** QMMM simulations, MD, POLARIZED MM, catalysis, CPMD-GULP

## Abstract

We report a quantum mechanics/polarizable–molecular mechanics (QM/p–MM) potential based molecular dynamics (MD) technique where the core–shell (or the Drude) type polarizable MM force field is interfaced with the plane-wave density functional theory based QM force field which allows Car–Parrinello MD for the QM subsystem. In the QM/p-MM Lagrangian proposed here, the shell (or the Drude) MM variables are treated as extended degrees of freedom along with the Kohn–Sham (KS) orbitals describing the QM wavefunction. The shell and the KS orbital degrees of freedom are then adiabatically decoupled from the nuclear degrees of freedom. In this respect, we also present here the Nosé–Hoover Chain thermostat implementation for the dynamical subsystems. Our approach is then used to investigate the effect of MM polarization on the QM/MM results. Especially, the consequence of MM polarization on reaction free energy barriers, defect formation energy, and structural and dynamical properties are investigated. A low point charge polarizable potential (p–MZHB) for pure siliceous systems is also reported here.

## 1. Introduction

Hybrid quantum mechanical/molecular mechanical (QM/MM) calculations offer a powerful way to bridge the length scales in a chemically complex system where a small region of the system of interest is treated by QM techniques, while the rest is described by computationally cheap MM force-fields. Widely used MM force fields employ a fixed point charge model for accounting the electrostatic interactions between MM atoms. The QM/MM implementations with fixed charge MM models enable polarization of QM charge density due to MM electrostatic potential. However, such approaches cannot take into account the polarization of MM atoms due to the QM electrostatic potential. Inclusion of polarization of MM atoms in QM/MM simulations demands usage of polarizable MM force fields, i.e., QM/polarized-MM (QM/p–MM) methods. It was reported that inclusion of polarization of MM atoms has significant effects on various properties (Bakowies and Thiel, 1996; Illingworth et al., 2006; Geerke et al., 2007; Lu and Zhang, 2008; Boulanger and Thiel, 2014), for instance, free energy barriers of chemical reactions are affected by about 10% (Lu and Zhang, 2008; Boulanger and Thiel, 2014).

The shell model (Dick and Overhauser, 1958) (or the Drude oscillator model) is widely used to describe polarization of MM atoms. Molecular dynamics (MD) simulations with the shell model based MM force fields can be carried out in two ways. In the conventional scheme, the position of the shells are minimized (Sangster and Dixon, 1976; Lindan and Gillan, 1993) at every MD step while keeping the core coordinates fixed. In an alternative scheme, the shells are treated as extended degrees of freedom and these are propagated classically to avoid minimization of their positions (Sprik, 1991; Mitchell and Fincham, 1993; Wilson and Madden, 1993) in the spirit of the Car–Parrinello MD method (Car and Parrinello, 1985). Often, the shell variables are assigned a mass smaller compared to that of the nuclear masses. Due to this reason, a smaller time step than used in a conventional MD is required for this approach. In practice, the shell temperature is kept close to 0 K, and most importantly, dynamics of shell degrees of freedom is made adiabatically decoupled from the rest of the system.

Different QM/MM MD schemes have been proposed to interfere the shell model with *ab initio* methods (Sulimov et al., 2002; Nasluzov et al., 2003; Woodcock et al., 2007; Geerke et al., 2007; Lu and Zhang, 2008; Lev et al., 2010; Boulanger and Thiel, 2012; Rowley and Roux, 2012; Boulanger and Thiel, 2014; Riahi and Rowley, 2014). Ideally, wavefunction of the QM subsystem and positions of shells are minimized at every MD step. In the approach by Lu and Zhang (2008) the positions of the shells were either minimized or updated only once in every MD step. The shell variables are treated as extended degrees of freedom in the QM/MM scheme proposed by Boulanger and Thiel (2012). Similar method was also reported by Rowley and Roux (2012). Recently Loco et al. (2016, 2017) have presented a QM/MM coupling method using the AMOEBA polarizable force fields. Here, SCF (self consistent field) calculations were carried out for the induced dipoles, while either SCF or the extended Lagrangian variant of Born-Oppenheimer MD (Niklasson et al., 2009) technique was employed for the wavefunction update. Incorporating the shell model in the extended Lagrangian scheme for Car–Parrinello MD within a QM/MM implementation is, however, not straightforward, and has not been attempted before, to the best of our knowledge.

In this paper we present an extended Lagrangian scheme to carry out Car–Parrinello MD for the QM subsystem which is coupled to a polarizable shell model based MM force field. First, we discuss the theory and the technical details of our method. Next, the implementation is validated by taking a system of water cluster composed of five water molecules. A new polarizable MM potential for silica with low point charges is then developed. Using our implementation and this new force-field for silica, we study three problems: (a) Oxygen vacancy in α–cristobalite silica; (b) Hydrogenation of ethene catalyzed by Rh cluster supported in Y–zeolite; (c) Proton exchange between methane and H–ZSM–5 zeolite.

## 2. Theory

### 2.1. Formulation of the extended lagrangian QM/p–MM method

The Lagrangian for the conventional QM/MM Car–Parrinello MD simulation is,

(1) $$\begin{array}{l} L_{\text{CP}/\text{QMMM}}\left(\text{R},\dot{\text{R}},\phi ,\dot{\phi }\right)=\underset{I}{\sum }\frac{1}{2}M_{I}{\dot{\text{R}}}_{I}^{2}+\underset{i}{\sum }\frac{1}{2}\mu \langle \dot{\phi_{i}}|\dot{\phi_{i}}\rangle \\ -E_{\text{KS}}\left(\text{R},\phi \right)-E_{\text{MM}}\left(\text{R}\right)-E_{\text{QM}/\text{MM}}\left(\text{R},\phi \right)\\ +\underset{i,j}{\sum }\Lambda_{ij}\left(\langle \phi_{i}|\phi_{j}\rangle -\delta_{ij}\right), \end{array}$$

where {*M*_*I*_} and {μ_*i*_} are the masses of the ionic and the orbital degrees of freedom, respectively, and {**R**_*I*_} and {ϕ_*i*_} are the nuclear coordinates (in Cartesian) and the Kohn–Sham orbitals, respectively. Here, *E*_KS_, *E*_MM_, and *E*_QM/MM_ are the energy of the QM subsystem, the energy of the MM subsystem and the energy due to QM-MM non-bonding interactions, respectively. The last term in the Lagrangian invokes orthonormality constraints during the classical evolution of the Kohn–Sham orbitals (Marx and Hutter, 2009). For details of this implementation, see Laio et al. (2002) and Sahoo and Nair (2016).

In the case of QM/p-MM implementation, MM polarization is included by augmenting the degrees of freedom by the shells {**r**_*k*_}, which are connected to a selected set of polarizable atoms P. We propose the QM/p-MM Lagrangian,

(2) $$\begin{array}{l} L_{\text{CP}/\text{Shell}}\left(\text{R},\dot{\text{R}},\text{r},\dot{\text{r}},\phi ,\dot{\phi }\right)=\underset{I}{\sum }\frac{1}{2}M_{I}{\dot{\text{R}}}_{I}^{2}+\underset{i}{\sum }\frac{1}{2}\mu \langle \dot{\phi_{i}}|\dot{\phi_{i}}\rangle +\overset{n_{\text{s}}}{\underset{k\in P}{\sum }}\frac{1}{2}m_{k}{\dot{\text{r}}}_{k}^{2}\\ -E_{\text{KS}}\left(\text{R},\phi \right)-E_{\text{MM}}\left(\text{R},\text{r}\right)-E_{\text{QM}/\text{MM}}\left(\text{R},\text{r},\phi \right)\\ +\underset{i,j}{\sum }\Lambda_{ij}\left(\langle \phi_{i}|\phi_{j}\rangle -\delta_{ij}\right), \end{array}$$

where, *n*_s_ is the number of shells, and *m*_*k*_ is the fictitious mass of a shell *k*. Also,

(3) $$\begin{array}{l} E_{\text{MM}}\left(\text{R},\text{r}\right)=E_{\text{b}}\left(\text{R},\text{r}\right)+E_{\text{nb}}\left(\text{R},\text{r}\right)+\overset{n_{\text{s}}}{\underset{k}{\sum }}\frac{1}{2}\kappa_{k}s_{k}^{2}, \end{array}$$

where

$$s_{k}=|{\text{s}}_{k}|=|{\text{R}}_{I}-{\text{r}}_{k}|,$$

having the shell *k* harmonically bound to the core atom I∈P. Here *E*_b_ refers to the sum of all the bonding terms in the MM potential, which is conventionally defined over the shell variables. The total non–bonding interaction energy, *E*_nb_, is the sum of the dispersive and the electrostatic interactions within the MM subsystem. The dispersive interactions are defined over the shell degrees of freedom, while the electrostatic interactions span over the cores and the shells degrees of freedom. Further,

(4) $$\begin{array}{l} E_{\text{QM}/\text{MM}}\left(\text{R},\text{r}\right)=E_{\text{b}}^{\prime }\left(\text{R},\text{r}\right)+E_{\text{vdw}}^{\prime }\left(\text{R},\text{r}\right)-\underset{I}{\sum }q_{I}^{\text{c}}\int d\bar{\text{r}}\rho \left(\bar{\text{r}}\right)\frac{1}{\left|{\text{R}}_{I}-\bar{\text{r}}\right|}\\ -\underset{k}{\sum }q_{k}^{\text{s}}\int d\bar{\text{r}}\rho \left(\bar{\text{r}}\right)\frac{1}{\left|{\text{r}}_{k}-\bar{\text{r}}\right|}, \end{array}$$

where $E_{\text{b}}^{\prime }$ and $E_{\text{vdw}}^{\prime }$ are the energy contributions due to the bonding and the dispersive interactions between the QM and the MM atoms, respectively. The last two terms in the above equation account for the electrostatic interaction between the point charges of the core ($\left\{q_{I}^{c}\right\}$) and the shell ($\left\{q_{k}^{s}\right\}$) degrees of freedom with the electronic density $\rho \left(\bar{\text{r}}\right)$, respectively. Electrostatic interactions are computed in the real space with the modified Coulomb kernel as in Laio et al. (2002).

For the success of this approach it is crucial that the Lagrangian in Equation (2) leads to dynamics close to that on the Born–Oppenheimer surface. This is taken care by starting the MD simulations with the optimized {ϕ} and {**r**}, while maintaining the temperatures of the orbitals (*T*_ϕ_) and the shells (*T*_s_) degrees of freedom close to zero, considering

(5) $$\begin{array}{l} \underset{\left\{\phi ,\text{r}\right\}}{\min}\underset{T_{\phi },T_{\text{s}}\to 0}{\lim}L_{\text{CP}/\text{Shell}}\left(\text{R},\dot{\text{R}},\text{r},\dot{r},\phi ,\dot{\phi }\right)\to L\left(\text{R},\dot{\text{R}}\right). \end{array}$$

The physical temperature (*T*_phys_) is defined as,

(6) $$\begin{array}{l} T_{\text{phys}}=\frac{1}{N_{\text{f}}k_{\text{B}}}\underset{I}{\sum }M_{I}{\dot{\text{S}}}_{I}^{2}, \end{array}$$

while the shell temperature is defined as,

(7) $$\begin{array}{l} T_{\text{s}}=\frac{1}{3n_{\text{s}}k_{\text{B}}}\overset{n_{\text{s}}}{\underset{k}{\sum }}{\bar{m}}_{k}{\dot{\text{s}}}_{k}^{2}. \end{array}$$

In the above equations, ${\dot{\text{S}}}_{I}$ is the velocity of the center of mass of a core–shell pair (*I, k*), and ${\dot{\text{s}}}_{k}$ is the relative velocity of the shell *k* connected to a core atom *I*. Here,

$${\text{S}}_{I}=\frac{1}{M_{I}}\left(M_{I}{\text{R}}_{I}+m_{k}{\text{r}}_{k}\right),$$

$M_{I}$ and ${\bar{m}}_{k}$ are the total mass of a core–shell pair (*I, k*), and the reduced mass of a shell *k*, respectively:

$$M_{I}=M_{I}+m_{k}$$

$${\bar{m}}_{k}=\frac{M_{I}m_{k}}{M_{I}+m_{k}}$$

In the above, *k*_B_ is the Boltzmann constant, *N*_f_ is the total nuclear degrees of freedom and *n*_s_ is the total number of shell variables, respectively. We have implemented this method in the CPMD/GULP QM/MM interface program, as developed in Sahoo and Nair (2016), where the plane wave density functional theory (DFT) based CPMD (CPMD, 132) code is interfaced with the MM based GULP (Gale, 1997) program.

At this stage, the following points are noted:

1. Both the orbital and the shell degrees of freedom have to be adiabatically separated from the nuclear degrees of freedom. While the nuclei could be kept hot, both the orbitals and the shells variables should remain cold throughout the dynamics.
2. As per Equation (5), it is not critical to have an adiabatic separation between shell and orbital degrees of freedom, provided that both sets of variables remain cold.

Accordingly, we have strategized the application of our implementation. The time step of integration and the masses of both shell and orbital degrees of freedom have to be chosen such that adiabatic separation between the nuclear subsystem and the subsystem containing shells and orbitals is maintained. We choose *m*_*k*_ = μ (i.e., the masses of the shell and the orbital degrees of freedom are taken to be the same), which allows us to choose the same time step for integrating the equations of motion for all the subsystems.

### 2.2. Implementation of Nosé–Hoover chain thermostat for shell dynamics

For obtaining stable dynamics and to achieve a canonical ensemble, it is crucial to couple the dynamical subsystems with thermostats. We have implemented three separate sets of Nosé–Hoover Chain (NHC) thermostats (Martyna et al., 1992). The system temperature is maintained at *T*_phys_ using one set of NHC thermostats whereas the shell and the orbital variables are maintained close to 0 K using two separate thermostats. We coupled the nuclear and the shell NHC thermostats to the center of mass motion and the relative motion of the core-shell pairs, and the corresponding equations of motion are given by,

$$\begin{array}{l} M_{I}{\ddot{\text{S}}}_{I}={\text{F}}_{I}^{\left(\text{S}\right)}-M_{I}{\dot{\text{S}}}_{I}\frac{p_{\eta_{1}}}{q_{1}}\\ {ṗ}_{\eta_{1}}=\underset{I}{\sum }M_{I}{\dot{\text{S}}}_{I}^{2}-N_{\text{f}}k_{\text{B}}T_{\text{phys}}-\frac{p_{\eta_{2}}}{q_{2}}p_{\eta_{1}}\\ {ṗ}_{\eta_{j}}=\frac{p_{\eta_{j-1}}^{2}}{q_{j-1}}-k_{\text{B}}T_{\text{phys}}-\frac{p_{\eta_{j+1}}}{q_{j+1}}p_{\eta_{j}},j=2,\cdots \;,n_{\text{c}}-1,\\ {ṗ}_{\eta_{j}}=\frac{p_{\eta_{j-1}}^{2}}{q_{j-1}}-k_{\text{B}}T_{\text{phys}},j=n_{\text{c}},\\ {\dot{\eta }}_{j}=\frac{p_{\eta_{j}}}{q_{j}},j=1,\cdots \;,n_{\text{c}}. \end{array}$$

Here ${\text{F}}_{I}^{\left(S\right)}$ is the force acting on the center of mass coordinates **S**_*I*_, {*p*_η_*i*__} and {*q*_*i*_} are the momentum and the masses of the thermostat variables η. Number of thermostat variables, *n*_c_, is chosen to be more than one.

In order to thermostat the shell dynamics, we write the shell equations of motion in relative coordinates as,

$$\begin{array}{l} {\bar{m}}_{k}{\ddot{\text{s}}}_{k}={\text{f}}_{k}^{\left(s\right)}-{\bar{m}}_{k}{\dot{\text{s}}}_{k}\frac{p_{\eta_{1}^{*}}}{q_{1}^{*}}\\ {ṗ}_{\eta_{1}^{*}}=\underset{k}{\sum }{\bar{m}}_{k}{\dot{\text{s}}}_{k}^{2}-3n_{\text{s}}k_{\text{B}}T_{\text{s}}-\frac{p_{\eta_{2}^{*}}}{q_{2}^{*}}p_{\eta_{1}^{*}}\\ {ṗ}_{\eta_{j}^{*}}=\frac{p_{\eta_{j-1}^{*}}^{2}}{q_{j-1}^{*}}-k_{\text{B}}T_{\text{s}}-\frac{p_{\eta_{j+1}^{*}}}{q_{j+1}^{*}}p_{\eta_{j}^{*}},j=2,\cdots \;,n_{\text{c}}^{*}-1,\\ {ṗ}_{\eta_{j}^{*}}=\frac{p_{\eta_{j-1}^{*}}^{2}}{q_{j-1}^{*}}-k_{\text{B}}T_{\text{s}},j=n_{\text{c}}^{*},\\ {\dot{\eta }}_{j}^{*}=\frac{p_{\eta_{j}}}{q_{j}^{*}},j=1,2,\cdots \;,n_{\text{c}}^{*}. \end{array}$$

Here ${\text{f}}_{k}^{\left(\text{s}\right)}$ is the force acting on the relative coordinate **s**_*k*_ of a core–shell pair (*I, k*). The thermostat variables for the relative motion are $\{\eta_{j}^{*}$}, having masses $\left\{q_{j}^{*}\right\}$, and their conjugate momenta are given by $\left\{p_{\eta_{j}^{*}}\right\}$. In our practical implementation, we transform the Cartesian coordinates of a core–shell pair to the corresponding relative and the center of mass coordinates at every MD steps. Transformation of forces for a core–shell pair (*I, k*) is achieved by,

$$\begin{array}{l} {\text{F}}_{I}^{\left(S\right)}={\text{F}}_{I}+{\text{f}}_{k}^{\left(r\right)},\\ {\text{f}}_{I}^{\left(s\right)}=\frac{1}{M_{I}}\left(m_{k}{\text{F}}_{I}-M_{I}{\text{f}}_{k}^{\left(r\right)}\right). \end{array}$$

Here, **F**_*I*_ and ${\text{f}}_{k}^{\left(r\right)}$ are the Cartesian forces on an atom (or core) *I* and on a shell *k* connected to core *I*, respectively. A similar coordinate transformation from cartesian to normal mode and vice–versa was reported by Marx et al. (1999) and can be also found in the work of Lamoureux and Roux (2003).

## 3. Low point charge polarizable force field for silica

Herein, the previously reported low point charge potential (MZHB) for silica (Sahoo and Nair, 2015) is further extended to include polarization of O atoms using the core shell model. This new potential is termed as p–MZHB hereafter. We have re-parameterized *k*_θ_ of O–Si–O and Si–O–Si angles and optimized the core-shell coupling parameters {κ_*k*_}. During the parameterization, point charges of the core and the shell of O atoms were allowed to vary, while their sum was fixed to −0.35 *e*. The re-parameterization was done using the GULP (Gale, 1997) program to reproduce the experimental structure of α–Quartz (Jorgensen, 1978). The final set of parameters are given in Table 1. The detailed validation of the p–MZHB potential is discussed in the Supporting Information.

**Table 1:** The p–MZHB force field parameters for siliceous zeolites.

	***k*_***r***_(eV/Å^2^)**	***r*_**0**_ (Å)**
Si–O	23.3	1.62
	*k*_θ_**(eV/rad**^2^**)**	θ_**0**_ **(**˙**)**
O–Si–O	6.061057	109.4
Si–O–Si	1.766554	149.8
	**Species**	**Charge (***e***)**
Si	core	0.70
O	core	1.387258
O	shell	-1.737258
	**Lennard–Jones**	
	ε(eV)	σ^0^(Å)
Si	0.00864	2.200
O	0.00324	1.770
	Species	κ(eV/Å^2^)
	O	99.4732

*For the cross Lennard–Jones parameters, the Lorentz–Berthelot (Leach, 2010; Schlick, 2010) combination rule is applied*.

## 4. Results and discussion

### 4.1. Validation of the implementation in CPMD/GULP interface program

**Figure 1:**
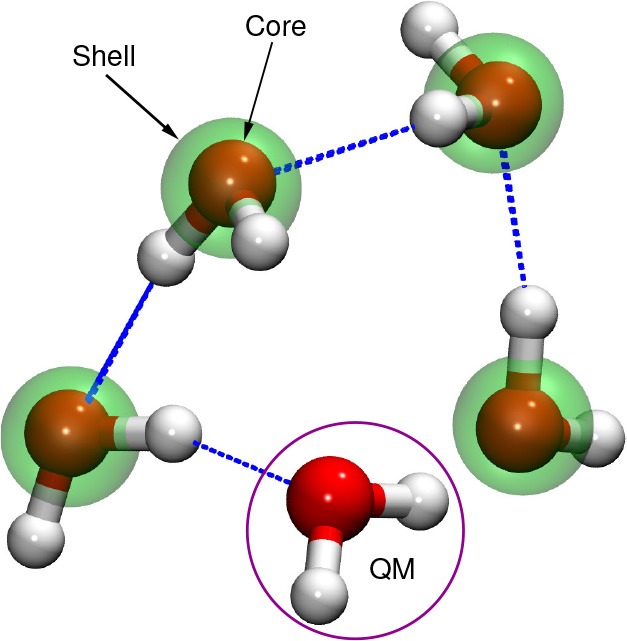
The system of five water molecules used for validating the QM/p–MM implementation. The QM water molecule is highlighted by a circle. The core and the shell of O atoms are indicated by red spheres and green transparent spheres, respectively. H atoms are in white color.

Total energy conservation during MD runs is tested to validate the method and the implementation. For the benchmarking purpose, we used 1H_2_O(QM)+4H_2_O(p–MM) system (Figure 1), and carried out *NVE* MD runs for 36 ps using DFT/PBE (Perdew et al., 1996) to describe the QM subsystem and polarizable de Leeuw–Parker (de Leeuw and Parker, 1998) potential to treat the MM subsystem; see Supporting Information for other technical details. In this MM potential, shells are added on the oxygen atoms of the water molecules.

**Figure 2:**
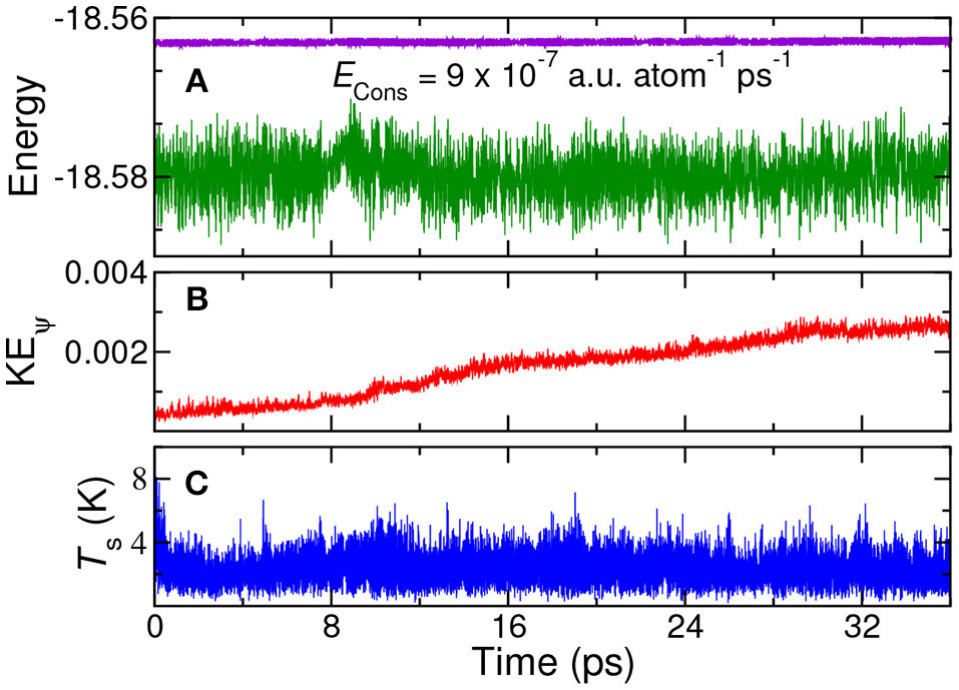
*NVE* MD simulation using QM/p–MM implementation for 1H_2_O (QM) + 4H_2_O (p–MM) system: **(A)** total energy (violet) and potential energy (green) **(B)** orbital kinetic energy, and **(C)** shell temperature. All the energies are in Hartree unit and temperature is in Kelvin. *E*_Cons_ is the drift in total energy per atom per ps.

The total energy, the orbital kinetic energy and the shell temperature of the system were monitored; see Figure 2. The drift in total energy is only of the order of 10^−7^ a.u. atom^−1^ ps^−1^, indicating that the total energy conservation is fairly good. The plot of kinetic energy of orbitals shows only a small long–time drift, while the temperature of the shell variables are maintained at low temperature. Such small drifts in kinetic energy could be controlled by connecting the orbital degrees of freedom with a thermostat, as demonstrated below.

**Figure 3:**
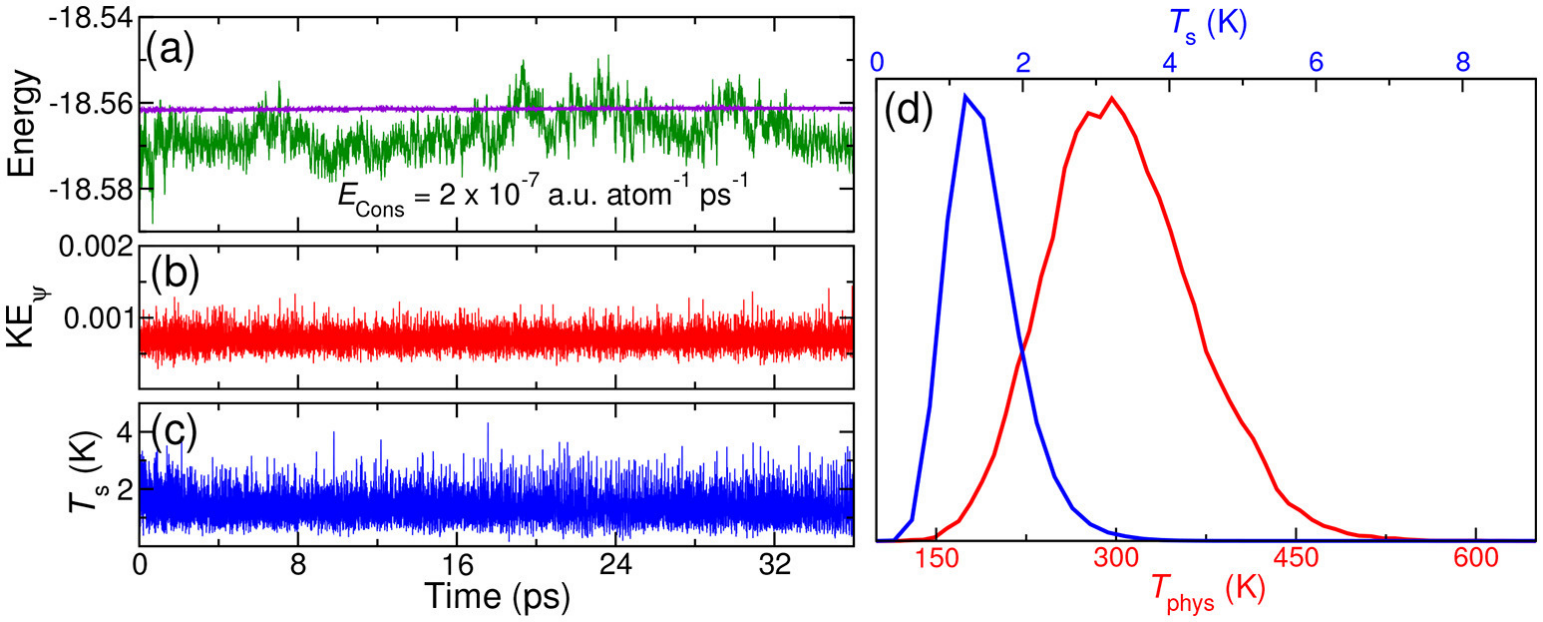
*NVT* MD simulation using QM/p–MM implementation for 1H_2_O (QM) + 4H_2_O (p–MM) system: **(A)** conserved energy (violet) and potential energy (green) **(B)** orbital kinetic energy **(C)** shell temperature *T*_s_ **(D)** distributions of physical temperature *T*_phys_ (red line with lower X–axis) and shell temperature *T*_s_ (blue line with upper X-axis). All the energies are in Hartree unit and the temperature is in Kelvin.

As next, we repeated the same simulation, but in the *NVT* ensemble. Here, three thermostats were added on the nuclear, the orbitals and the shells degrees of freedom, and these were thermostated to 300 K (*T*_phys_, 0.0007 Hartree and 1 K, respectively). Since the NHC thermostat posseses a conserved quantity, drift in this conserved quantity allows us to verify our implementation further. The drift in the conserved quantity is only of the order of 10^−7^ a.u. atom^−1^ ps^−1^, confirming the correctness of our implementation (see Figure 3A). The orbital kinetic energy and the shell temperature (see Figure 3B–D) plots clearly show that the dynamics of the extended variables is stable and is well thermostated.

### 4.2. Benchmark studies using α–cristobalite silica

**Figure 4:**
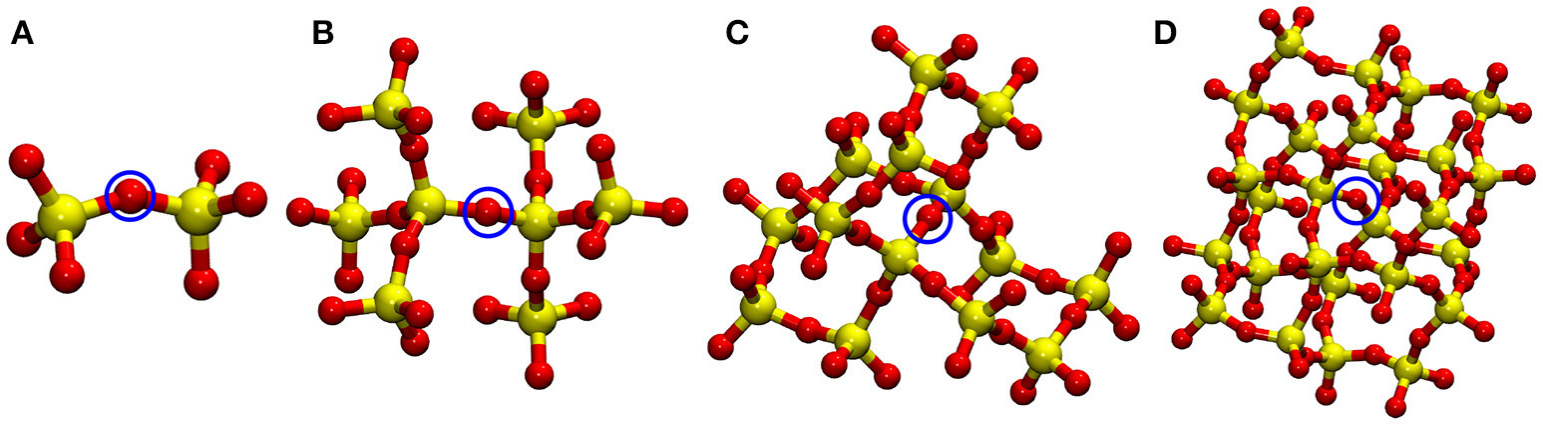
QM subsystems used in the four QM/MM calculations of oxygen vacancy in α–Cristobalite: **(A)** 2T (Si_2_O_7_), **(B)** 8T (Si_8_O_25_), **(C)** 14T (Si_14_O_40_), and **(D)** 26T (Si_26_O_67_). In the vacancy calculations, the highlighted (by blue circle) O atom was removed from its lattice position. Note that MM atoms are not shown here. Atom colors: Si (yellow), O (red).

To further benchmark the performance of the developed method, we carried out MD simulation of α–cristobalite silica in the *NVT* ensemble at 300 K. Here we use the DFT/PBE (Perdew et al., 1996) level of theory for the QM part and p-MZHB potential for the MM part. We used a supercell of 8 × 8 × 8 (Si_2048_ O_4096_) for QM/p–MM calculations. Optimized structure and lattice parameters using the p–MZHB MM potential were used here. Multiple QM/p–MM calculations were carried out with different QM sizes: 2T (Si_2_O_7_), 8T (Si_8_O_25_), 14T (Si_14_O_40_), and 26T (Si_26_O_67_), where T stands for SiO_4_ tetrahedral unit (see Figure 4).

The structure (inner QM atoms only) obtained from QM/p–MM simulation was compared with QM data (“all–QM”) and MM data (“all–MM”) data; see Table 2. Difference between “all–QM” and QM/p–MM data for Si–O bond length is only 0.01 Å, O–Si–O angle is only 0.2° and Si–O–Si angle is only 0.8°. However, structures near the boundary are deviating more from the “all–QM” data; see Supporting Information. This is expected due to the boundary effects in QM/MM calculations (as also seen in Sahoo and Nair, 2016).

**Table 2:** The average value of Si–O bond length (Å), O–Si–O, and Si–O–Si angles (°) of α–cristobalite computed from MD simulations in *NVT* ensemble at 300 K using p–MZHB, MZHB (Sahoo and Nair, 2015), QM/p–MM (14T), and QM potentials.

	**“all–MM”^a^**	**“all–QM”^b^**	**QM/p–MM^c^ (14T)**	**Expt**.
Si–O bond length	1.61(±0.03)	1.63(±0.03)	1.62(±0.03)	1.60(3)
O–Si–O angle	109.4(±3.1)	109.2(±4.0)	109.4(±3.8)	108.2–111.4
Si–O–Si angle	147.7(±4.5)	142.2(±6.3)	143.0(±5.7)	146.4(9)

*These results are also compared with experimental data (Downs and Palmer, 1994). For details see text*.

a *Using p–MZHB MM potentials*.

b *Using PBE density functional*.

c *Using PBE density functional and p–MZHB MM force–field*.

Next, the vacancy formation energy, (Δ*E*_f_) in α–cristobalite silica was then computed, as

(8) $$\begin{array}{l} \Delta E_{\text{f}}=\frac{x}{2}\left[E\left({\text{O}}_{2}\right)+E_{\text{diss}}\left({\text{O}}_{2}\right)\right]+E\left({\text{SiO}}_{2-x}\right)-E\left(\text{Si}{\text{O}}_{2}\right) \end{array}$$

where *E*(O_2_), *E*_diss_(O_2_), *E*(SiO_2−*x*_), and, *E*(SiO_2_) are the energies of O_2_ molecule (in the triplet electronic ground state), the dissociation energy of O_2_ molecule, the energy of bulk silica with oxygen vacancy, and the energy of pure bulk silica, respectively. *E*(O_2_) was computed from “all–QM” calculations whereas *E*(SiO_2−*x*_) and *E*(SiO_2_) were computed either by “all–QM” or QM/p–MM calculations. *E*_diss_(O_2_) = 5.16eV was taken from the available experimental data (Lide, 2005). Δ*E*_f_ values computed from “all–QM” calculations with varying supercell sizes are listed in Table 3. The converged value of Δ*E*_f_ (w.r.t supercell size) is 8.73 eV.

**Table 3:** Δ*E*_f_ computed from the single point energy calculations using “all–QM” with PBE density functional.

**Supercell**	***ΔE*_f_ (eV)**
2 × 2 × 2	8.68
3 × 3 × 2	8.73
3 × 3 × 3	8.73

Δ*E*_f_ values computed from QM/p–MM calculations listed in Table 3, show that Δ*E*_f_ is nearly converged to 8.77 eV which is in excellent agreement with the “all–QM” data. However, it may be noted that the values predicted by QM/p–MM calculations are sensitive to the size of the QM subsystem. The Δ*E*_f_ value of 9.43 eV using 2T site is a very poor estimate compared with the “all–QM” data. Clearly, the computed value converges close to “all–QM” data with increase in the size of the QM subsystem (see Table 4).

**Table 4:** Δ*E*_f_ computed from single point energy calculations using QM/p–MM potential.

**Supercell**	**QM sites**	***ΔE*_f_ (eV)**
8 × 8 × 8	2T	9.43
	8T	8.76
	14T	8.78
	26T	8.77
9 × 9 × 9	8T	8.76
	14T	8.77
	26T	8.77

**Figure 5:**
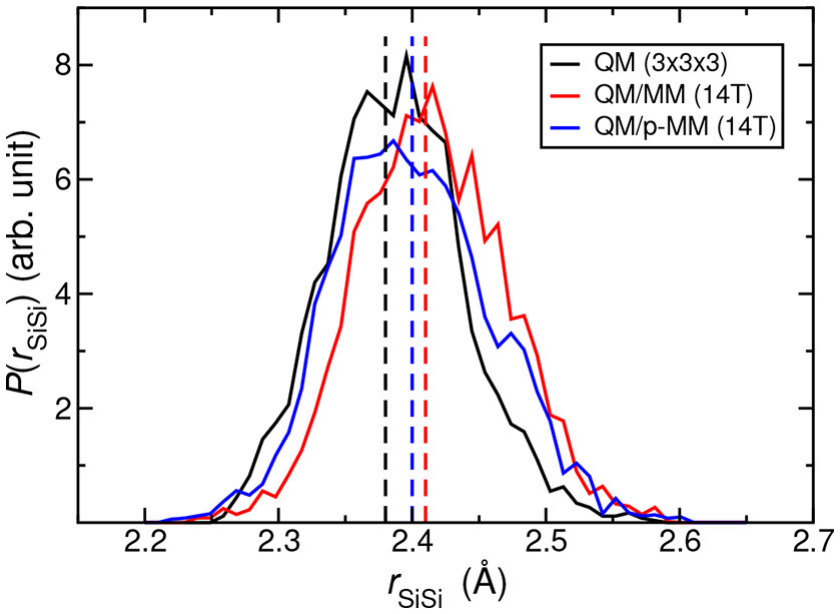
Probability distribution of *r*_SiSi_ distance obtained from the MD simulations at 300 K using “all–QM” (3 × 3 × 3 supercell), QM/MM (14T) and QM/p–MM (14T) potentials. The vertical lines show the corresponding average values.

The bond length distribution of the Si–Si bond (*r*_SiSi_) at the defect site is then computed from the *NVT* trajectory using “all–QM” (using 3 × 3 × 3 supercell), QM/MM, and QM/p–MM potentials; see Figure 5. The average *r*_SiSi_ value using “all–QM” potential is 2.38 Å. Using the non-polarized MZHB QM/MM simulations, we obtained a slightly increased value of 2.41 Å, while this was 2.40 Å in the polarized case. Overall, Figure 5 shows that the mean and the standard deviation of *r*_SiSi_ from QM/p–MM MD agree better to the “all–QM” MD results than that from the QM/MM MD using the non–polarizable MM force–field.

### 4.3. Application: hydrogenation of ethene catalyzed by Rh clusters supported in Y–zeolite

In order to investigate the effect of polarization of MM atoms on the free energy barriers, we revisit the study of hydrogenation of ethene catalyzed by Rh clusters supported in Y–zeolites, as in our previous study (Sahoo and Nair, 2016). Based on the experimental results from the Gates group (Liang and Gates, 2008) and the previous study (Sahoo and Nair, 2016), we choose the hydrogenated cluster Rh_3_H_7_ in Y–zeolite as the catalyst model. In order to model Y–zeolite, a supercell of size 2 × 2 × 2 of pure siliceous Y–zeolite (Si_1536_O_3072_) was taken. The metal cluster, its ligands, and the metal–coordinated T25 site of the Y–zeolite were treated in the QM region (at the level of DFT/PBE+D2; Perdew et al., 1996; Grimme, 2006), while the rest of the zeolite was treated using MZHB or p–MZHB MM potentials.

**Figure 6:**
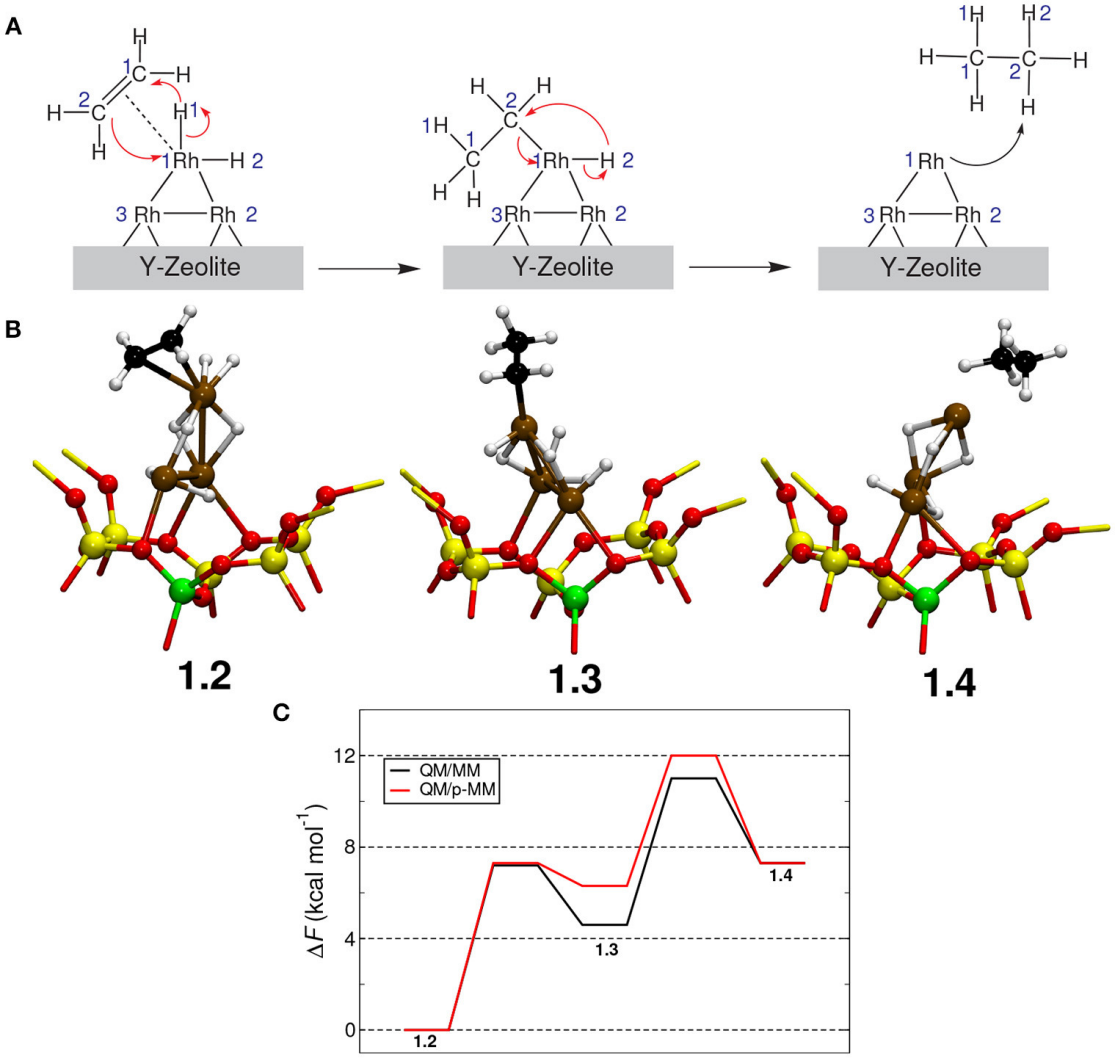
**(A)** Mechanism of hydrogenation of ethene catalyzed by Rh_3_H_7_/Y. **(B)** Snapshots of the reactant(**1.2**), the intermediate(**1.3**) and the product(**1.4**). Atom colors: Rh–ochre, Si–yellow, Al–green, O–red, C–black, and H–white. **(C)** Free energy profiles computed from the QM/MM and the QM/p–MM metadynamics simulations are given.

The free energy profile for ethene hydrogenation was computed employing metadynamics (Laio and Parrinello, 2002) using the QM/MM and QM/p–MM implementations; see Figure 6 for the mechanism of the reaction studied here. For the details of the metadynamics simulation setup, see Supporting Information. In the metadynamics simulations using QM/MM and QM/p–MM methods, we observed the same reaction mechanism. Here, one of the hydrogen atoms from Rh_3_H_7_ cluster moved to one of the C atoms of ethene forming an intermediate **1.3**, which further reacted with a hydrogen atom on the Rh cluster to form ethane (**1.4**).

Interestingly, we observed differences of the order of 1 kcal mol^−1^ only in the free energy barriers computed from QM/p-MM and QM/MM computations; see Figure 6C. The main difference is in the stability of the ethyl intermediate **1.3** and on the free energy barrier for **1.3**→**1.4**. However, the difference in free energy is only ~1 kcal mol^−1^ which is close to the error associated with the metadynamics simulation. Thus, we conclude that for this specific reaction, the effect of MM polarization in the free energy estimates and on the reaction mechanism are negligible.

### 4.4. Application: proton exchange between methane and H–ZSM–5 zeolite

**Figure 7:**
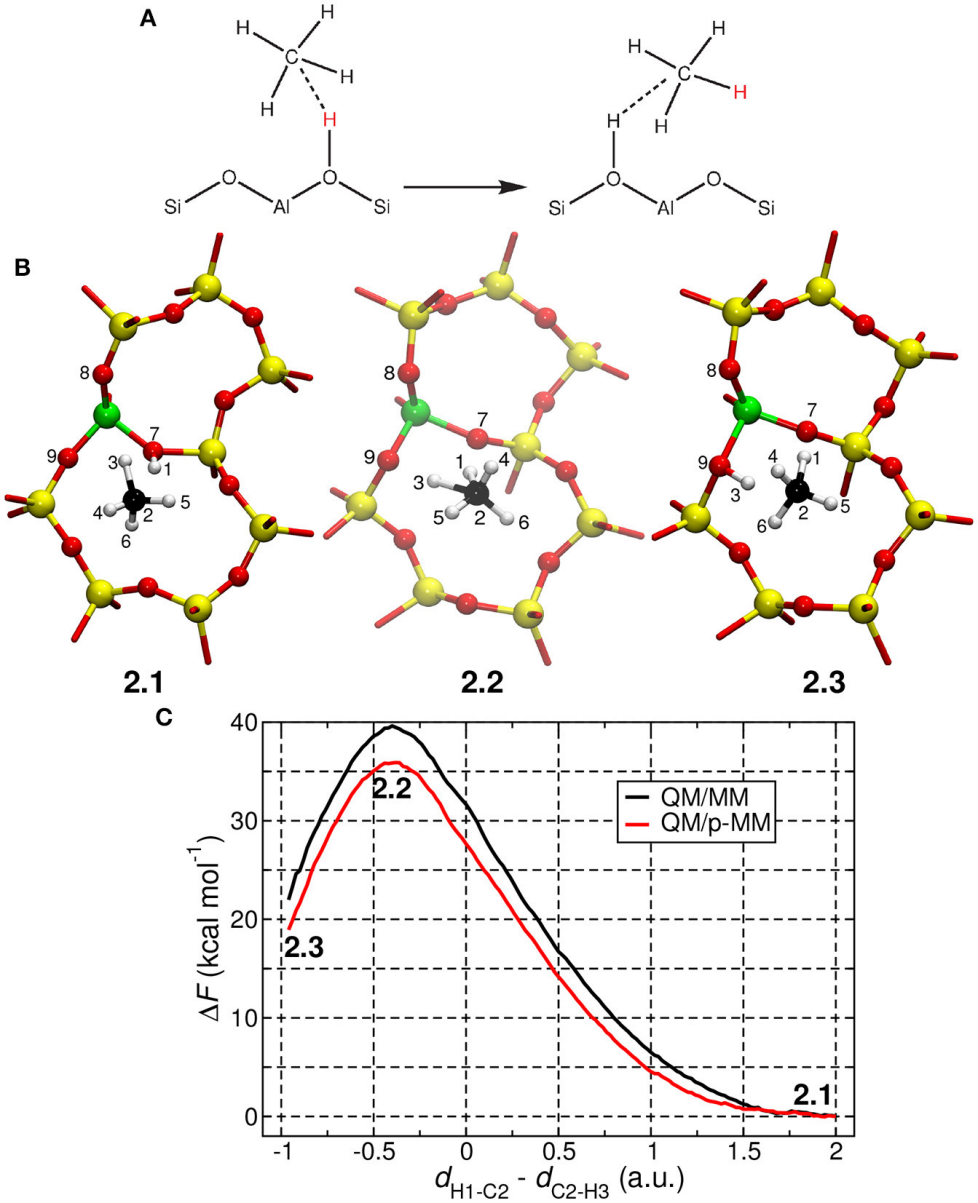
**(A)** Mechanism of the proton exchange reaction between a methane molecule and the H–ZSM–5 zeolite. **(B)** Snapshots of the reactant, transition state, and product states taken from QM/p–MM MD simulations. Here the atoms colors used are Si (yellow), Al (green), O (red), C (black), and H (white). **(C)** Free energy profiles constructed from TASS simulations are shown here. The free energy profiles were obtained by projecting the six–dimensional free energy surface on the CV, *d*_H1−C2_ − *d*_C2−H3_, which is the difference in the distances H1–C2 and C2-H3.

Methane activation is one of the important steps in various industrially relevant processes such as conversion of methane to higher hydrocarbons and methanol. In this process, the C–H bond, which is quite inert as evident from the high bond formation energy (>100 kcal mol^−1^), is activated for further functionalization. Of great importance, protonated zeolites, particularly H–ZSM–5, is known to activate the methane C–H bonds (Arzumanov et al., 2014; Chu et al., 2016). Here we look at the following chemical reaction (Figure 7A):

(9) $$\begin{array}{l} \text{H}-\text{Z}S\text{M}+{\text{H}}^{\prime }-{\text{CH}}_{3}\to {\text{H}}^{\prime }-\text{Z}S\text{M}+\text{H}-{\text{CH}}_{3}. \end{array}$$

One of the acidic protons in the H–ZSM–5 zeolite is exchanged with one of the protons in the methane molecule during this reaction. The mechanism for C–H activation in zeolites is previously studied in the literature (Vollmer and Truong, 2000; Zheng and Blowers, 2005; Truitt et al., 2006; Bučko et al., 2007; Gabrienko et al., 2011; Arzumanov et al., 2014; Tuma and Sauer, 2015; Chu et al., 2016). Two types of mechanisms are proposed for this reaction: (a) direct and (b) bimolecular. In the direct mechanism, hydrogen is exchanged through a carbonium ion intermediate, i.e., via the formation of the penta–coordinated carbocation. In the bimolecular mechanism, the alkane molecule dissociates one of H atoms to the zeolite framework, forming an alkoxyl intermediate (Truitt et al., 2006), which subsequently undergoes reactions with other alkane molecules. Chu et al. (2016) have reported from both experimental and theoretical studies that higher alkanes react through bimolecular mechanism whereas the lower alkanes react via the direct mechanistic route. Our interest here is the direct mechanism as it involves the formation of a charged reactive intermediate and we anticipate some effect of MM polarization in the free energy estimates.

We could successfully simulate the proton exchange reaction using the Temperature Accelerated Sliced Sampling (TASS) method (Awasthi and Nair, 2017). This method allowed us to explore a high–dimensional free energy landscape composed of five collective variables (CVs). More details about the CVs used here and other technical details of the TASS simulation are given in the Supporting Information. The computed free energy profiles (projected along one of the crucial CVs) are given in Figure 7C. In TASS+QM/MM and TASS+QM/p-MM simulations, the hydrogen exchange reaction was found to proceed through the formation of a carbonium ion (${\text{CH}}_{5}^{+}$; see structure **2.2** in Figure 7B). The free energy barrier (Δ*F*^‡^) for the reaction computed from QM/MM and QM/p-MM simulations are 39.5 and 36.0 kcal mol^−1^, respectively. As expected, we are observing significant difference in the free energy barriers when polarized MM force-field is used. It is also noted in passing that the free energy barrier computed here are close to the potential energy barriers computed for similar reactions in zeolites in Vollmer and Truong (2000), Zheng and Blowers (2005), Bučko et al. (2007), and Tuma and Sauer (2015), which are in the range 29–38 kcal mol^−1^.

## 5. Conclusions

An extended Lagrangian based implementation of QM/p–MM method that allows to perform conventional Car–Parrinello MD for the QM subsytem is presented here. In particular, we have discussed a combined scheme where the extended Lagrangian dynamics of the shell (or the Drude) variables are performed together with the Car–Parrinello dynamics of the KS orbitals. Inclusion of polarization does not increase the computational cost of the QM/MM Car–Parrinello MD simulations within our approach, mainly because we use the same time step as that of the conventional Car–Parrinello MD.

We find that, invoking polarization of MM atoms in QM/MM calculations only marginally improve the predictions of equilibrium structure and dynamics of non–charged systems. On the other hand, when the transition state is charged, free energy barriers are considerably affected on the inclusion of MM polarization. We believe that the methods and the strategies developed here for a QM/polarized–MM implementation will be useful to study more complex problems in catalysis, reactions in solid–liquid interfaces, crystallization etc.

Other than these, we also report here a new low point charge polarizable potential for silica, which is suitable for performing QM/MM calculations. The potential is shown to be performing well and is able to reproduce bulk structures of various silica polymorphs. The developed MM potential has simple and commonly used potential functions with fewer parameters, making it easy to use in various simulation packages such as GULP, DL_POLY (Todorov et al., 2006) and LAMMPS (Plimpton, 1995).

## Author contributions

NN has planned the work and SS have executed the implementation and computations. Both authors have analyzed the results and have written the paper.

### Conflict of interest statement

The authors declare that the research was conducted in the absence of any commercial or financial relationships that could be construed as a potential conflict of interest. The handling Editor and reviewer TH declared their involvement as co-editors in the Research Topic, and confirm the absence of any other collaboration.
