# Supplementary material for: Interfacing the Core-Shell or the Drude Polarizable Force Field With Car–Parrinello Molecular Dynamics for QM/MM Simulations
Source: Front Chem. 2018 Jul 10;6:275. doi: 10.3389/fchem.2018.00275 (PMC6048201; doi:10.3389/fchem.2018.00275)
Supplement: Supplementary file 1 [file Presentation_1.PDF]

Supporting Information for Interfacing the Core Shell or the Drude Polarizable Force Field with the Car–Parrinello Molecular Dynamics for QM/MM Simulations

Sudhir K. Sahoo and Nisanth N. Nair

Department of Chemistry, Indian Institute of Technology, Kanpur, India

1 Validation of the p–MZHB Potential

In order to validate the developed polarizable MZHB potential (p-MZHB), we computed the bulk structures of six different types of silica polymorphs using the p–MZHB potential and compared with the available experimental data and that computed using other force–fields; see Table SI 1 for the supercells used for these simulations. The initial structure of α–cristobalite was obtained from Ref. [1] , and that of the other pure siliceous zeolites were obtained from the zeolite database. [2] The optimized lattice parameters computed using p–MZHB and other potentials are listed in Table SI 3. The mean average error (MAE) and the maximum error (MaxE) were computed with respect to the available experimental structural data for quantifying the performance. Based on this, we conclude that the the overall performance (with MAE of 0.083 Å and MaxE of 0.239 Å) of the p–MZHB potential is quite satisfactory. On the other hand, it is clear that the potentials developed by Sanders et al. [3] and the MZHB potential previously developed by us[4] perform better than the currently developed p–MZHB potential.

Table SI 1: The SiO_2 supercells used for modeling various SiO_2 polymorphs to validate the p–MZHB potential.

Silica	Supercell size	Formula
Faujasite (FAU)	$1 \times 1 \times 1$	$Si_{192}O_{384}$
Chabazite (CHA)	$2 \times 2 \times 2$	$Si_{288}O_{576}$
Sodalite (SOD)	$3 \times 3 \times 3$	$Si_{324}O_{648}$
ZSM–5	$2 \times 2 \times 2$	$Si_{768}O_{1536}$
α-Quartz	$6 \times 6 \times 6$	$Si_{648}O_{1296}$
α-Cristobalite	$6 \times 6 \times 6$	$Si_{864}O_{1728}$

Then we carried out MM MD simulations of α–Cristobalite silica to further test the p–MZHB potential. MD simulations were carried out using the GULP program, [5] with a shell mass of 1468 a.u. (which is 5% of mass of O atom). NVT ensemble MD simulations were carried out at 300 K using the Nosé–Hoover thermostat [6] with the Nosé–Hoover parameter `qnose` (as defined in the GULP program [5]) set to 0.1. The MD time step for these simulations was taken as 0.1 fs.

Table SI 2: The p–MZHB force field parameters for siliceous zeolites. For the cross Lennard–Jones parameters, the Lorentz–Berthelot [7, 8] combination rule is applied. MZHB potential parameters are also given here for a comparison.

	p-MZHB		MZHB	
	k_r(eV/Å^2)	r_0 (Å)	k_r(eV/Å^2)	r_0 (Å)
Si–O	23.3	1.62	23.3	1.62
	k_θ(eV/rad^2)	θ_0 (°)	k_θ(eV/rad^2)	θ_0 (°)
O–Si–O	6.061057	109.4	6.800000	109.47
Si–O–Si	1.766554	149.8	2.220000	149.80
	Species	Charge (e)	Species	Charge (e)
Si	core	0.70	core	0.70
O	core	1.387258	core	-0.35
O	shell	-1.737258		
	Lennard–Jones		Lennard–Jones	
	ε(eV)	σ^0(Å)	ε(eV)	σ^0(Å)
Si	0.00864	2.200	0.00864	2.200
O	0.00324	1.770	0.00324	1.770
	Species	κ(eV/Å^2)		
	O	99.4732		

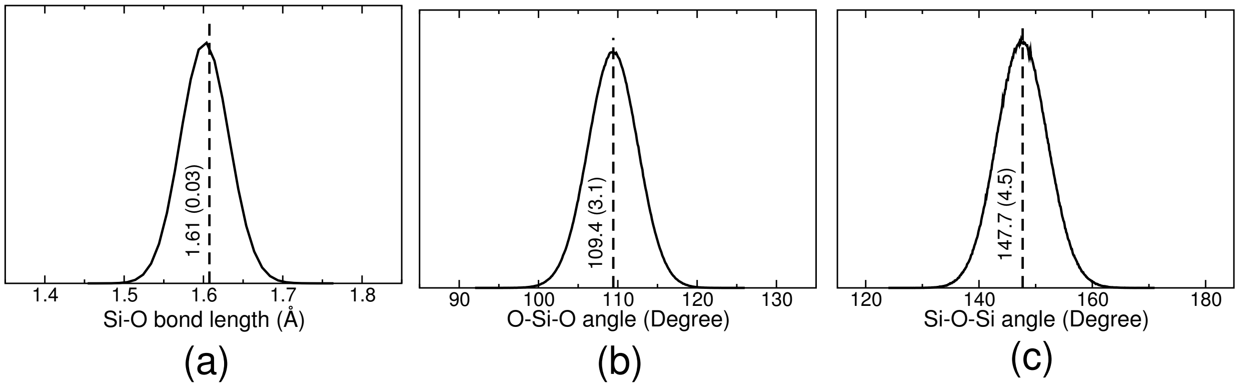

Figure SI 1: The probability distributions of Si–O bond length, O–Si–O and Si–O–Si angles of α–cristobalite silica computed from a canonical ensemble MD simulation at 300 K. Here, the vertical lines show the corresponding average values. Standard deviations are indicated in parentheses.

A separate MD simulation was carried out using periodic DFT (fully QM) potential. Here, a supercell of $3 \times 3 \times 3$ ($Si_{108}O_{216}$) was used to model the bulk α–cristobalite silica. The initial structure and lattice parameters were obtained from Ref. [1] The PBE [9] density functional was employed and the core electrons were replaced by Vanderbilt ultrasoft pseudopotentials. [10] Plane wave cutoff of 30 Ry was used to expand the Kohn–Sham orbitals. Car–Parrinello MD simulation was performed with a fictitious orbital mass of 600 a.u. and MD time step was set to 0.096 fs (4 a.u.). Temperature of the system was maintained at 300 K using NHC thermostat [11] with the NHC mass parameters assigned as 2500 and 10000 cm^{-1} for the nuclei and the orbitals, respectively.

Distributions of Si–O bond length and O–Si–O and Si–O–Si angles from MD simulations using the p–MZHB potential are compared with those obtained using the fully QM and the MZHB [4] potentials and the available experimental data.

The results of this study are given in Figure SI 1 and Table SI 4. Interestingly, the performance of the p–MZHB potential is very close to the MZHB potential and agree well with the experimental and all-QM results. The only noticeable difference is that the Si–O bond distance is overestimated by the p–MZHB potential by 0.01 Å, while the Si–O–Si angle is predicted better by the p–MZHB potential compared to the MZHB potential. On the other hand, both the MM potentials (p–MZHB & MZHB) performed better than the all-QM (DFT/PBE) calculations.

2 Computational Details of the QM/p–MM Calculations of $(H_2O)_5$ Water Cluster

The whole system (composing of all the five water molecules) was taken in a periodic cubic box of side 100.0 Å. The QM subsystem was taken in a cubic supercell of side 10.58 Å to simulate an isolated QM density for the QM water molecule.

Table SI 3: Comparison of optimized lattice parameters (in Å) for six different silica polymorphs obtained using various potentials and available experimental data. The mean average error (MAE) and the maximum error (MaxE) are computed with respect to the experimental values are also given.

Structure		Expt	p–MZHB	MZHB [4]	Sanders et al. [3]	Nasluzov et al. [12]	Schröder & Sauer [13]
FAU[a]	$a=b=c$	24.258	24.309	24.396	24.271	24.647	24.632
CHA[b]	$a=b$	13.529	13.606	13.639	13.549	13.729	13.712
	c	14.748	14.691	14.722	14.581	14.781	14.838
SOD[c]	$a=b=c$	8.836	8.902	8.907	8.840	8.927	8.951
ZSM–5[d]	a	20.087	20.070	20.171	20.135	20.275	20.425
	b	19.894	19.655	19.749	19.827	19.679	20.205
	c	13.372	13.173	13.264	13.406	13.301	13.634
α–Quartz[e]	$a=b$	4.915	4.978	5.000	4.866	4.877	4.988
	c	5.406	5.422	5.460	5.374	5.346	5.506
α–Cristobalite[f]	$a=b$	4.972	5.034	5.059	5.011	4.981	5.129
	c	6.922	6.853	6.919	7.064	6.833	7.266
MAE		–	0.083	0.083	0.056	0.126	0.213
MaxE		–	0.239	0.145	0.167	0.389	0.374

[a] Ref [14], [b] Ref [15], [c] Ref [16], [d] Ref [17], [e] Ref ,[18] and [f] Ref [19]

Table SI 4: The average value of Si–O bond length (Å), O–Si–O and Si–O–Si angles (°) of α–cristobalite computed from NVT MD simulations at 300 K using the p–MZHB, the MZHB [4], QM/p–MM (14T) and all-QM potentials. These results are also compared with the available experimental data. [19] For details see text.

	p–MZHB	MZHB	all-QM	QM/p–MM (14T)	Expt.
Si–O bond length	1.61(±0.03)	1.60(±0.03)	1.63(±0.03)	1.62(±0.03)	1.60(3)
O–Si–O angle	109.4(±3.1)	109.4(±2.9)	109.2(±4.0)	109.4(±3.8)	108.2–111.4
Si–O–Si angle	147.7(±4.5)	150.7(±4.2)	142.2(±6.3)	143.0(±5.7)	146.4(9)

Polarizable MM water molecules were using a flexible water model developed by de Leeuw and Parker [20], where the polarization of O atom is treated by the core–shell model.

PBE [9] exchange correlation functional was employed and a plane wave cutoff of 30 Ry was chosen. Core electrons were treated using the Vanderbilt ultrasoft pseudopotentials. [10]

The time step for integrating the equations of motion was 0.12 fs (5 a.u.). All other technical details of this simulation are the same as that mentioned in the previous section.

3 Computational Details of Benchmark Studies using α–Cristobalite

Two supercells of sizes $8 \times 8 \times 8$ (Si_{2048} O_{4096}) and $9 \times 9 \times 9$ (Si_{2916} O_{5832}) were considered for QM/p–MM calculations.; see also the main text. Multiple QM/p–MM calculations were carried out with different QM sizes: 2T (Si_2O_7), 8T (Si_8O_{25}), 14T ($Si_{14}O_{40}$) and 26T ($Si_{26}O_{67}$), where T stands for one SiO_4 tetrahedral unit. For the QM system sizes used in the QM/MM calculations, see Figure 4 in the main text. The QM part of the system was treated by plane–wave DFT using the PBE [9] exchange correlation functional. The MM part of the system was treated by the p–MZHB force–field. All other technical details are the same as in the previous section.

MD simulation of pure α–cristobalite using “all-QM”, QM/MM, QM/p-MM, and “all-MM” potentials were performed for 11, 12, 10 and 60 ps, respectively. For the case of α–cristobalite silica with defect, the MD simulations using “all-QM”, QM/MM, and QM/p-MM potentials were carried out for 13, 12, and 12 ps, respectively.

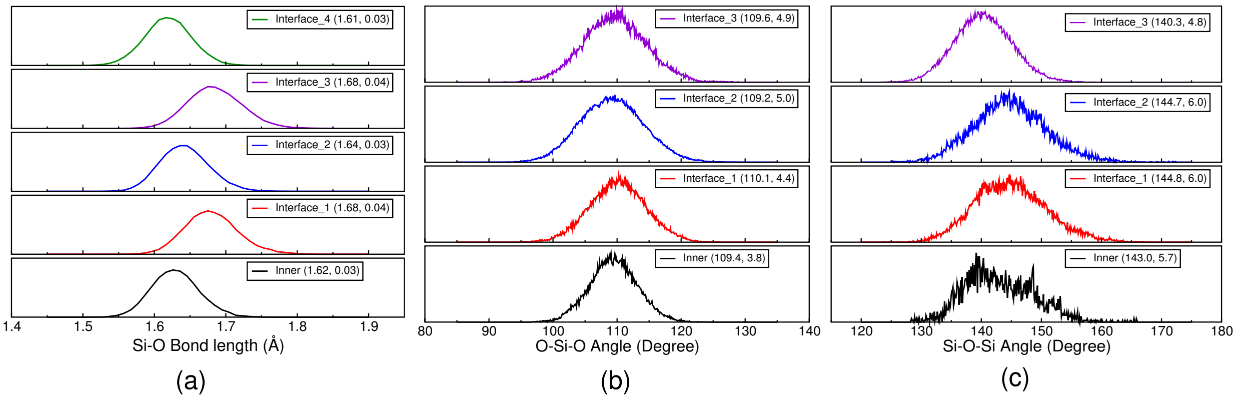

Figure SI 2: The distributions of (a) Si–O bond length ,(b) O–Si–O angle, and, (c) Si–O–Si angle for different regions (see Tables SI 5, SI 6, and, SI 7) from the QM/p–MM MD simulations at 300 K are shown. The corresponding average value with standard deviations are given in the brackets.

Table SI 5: The average values and the standard deviations of the Si–O bond length (Å) for various regions computed from the hybrid QM/p–MM MD in NVT ensemble at 300 K.

	Average	Type
Inner	1.62(0.03)	[Si–O]
Interface_1	1.68(0.04)	Si–[O–Si_{OH}]
Interface_2	1.64(0.03)	Si_{OH}–[O–Si_{OH}]
Interface_3	1.68(0.04)	[Si_{OH}– O_H]–Si_{MM}
Interface_4	1.61(0.03)	Si_{OH}–[O_H–Si_{MM}]

Table SI 6: The average values and standard deviations of O–Si–O angle (°) for various regions computed from the hybrid QM/p–MM MD in NVT ensemble at 300 K.

	Average	Type
Inner	109.4(3.8)	[O–Si–O]
Interface_1	110.1(4.4)	[O–Si_{OH}–O]
Interface_2	109.2(5.0)	[O–Si_{OH}–O_H]
Interface_3	109.6(4.9)	[O_H–Si_{OH}–O_H]

Table SI 7: The average values and standard deviations of Si–O–Si angle (°) for various regions computed from the hybrid QM/p–MM MD in NVT ensemble at 300 K.

	Average	Type
Inner	143.0(5.7)	[Si–O–Si]
Interface_1	144.8(6.0)	[Si_{OH}–O–Si_{OH}]
Interface_2	144.7(6.0)	[Si–O–Si_{OH}]
Interface_3	140.3(4.8)	[Si_{OH}–O_H–Si_{MM}]

4 Hydrogenation of Ethene Catalyzed by Rh Clusters Supported in Y–Zeolite

One of the central Si atoms in the QM regions of the Y–zeolite was replaced by an Al atom. The negative charge acquired due to this was balanced by the odd number of hydrogen atoms bound to Rh cluster, as treated in Ref. [21].

The QM region was treated in a cubic box of side 23.276 Å. The PBE density functional with D2 dispersion correction[9, 22] was chosen as the exchange correlation functional. MM part was treated using the p–MZHB or the MZHB potentials. All other technical details of QM/MM and MD simulations are the same as in the previous section.

Metadynamics [23] simulations were performed for simulating the reactions given in Figure 7a. Here, we used the extended Lagrangian variant of metadynamics [24] with a force constant of 2 a.u. for coupling collective variables (CV) with the corresponding auxiliary variables. A fictitious mass of 50 a.m.u. was chosen for these auxiliary variables. Spherical gaussian functions with a width of 0.05 (CV units) and a height of 0.6 kcal mol^{-1} was used to construct the bias potentials . An adaptive metadynamics bias update scheme is used where the update was performed only when the CVs were diffused more than 1.5 times the width of the Gaussian. In these runs, temperature of the auxiliary CV variables were kept close to 300 K by velocity scaling.

The list of CVs used for simulating this reaction is given in Table SI 8. We employed a coordination number type of CV between atoms of type A and atoms of type B, which is defined as,

$$C^{\text{tot}}[\text{A} - \text{B}] = \sum_{I \in \text{A}} \sum_{J \in \text{B}} \frac{1}{1 + (R_{IJ}/d^0)^6} \ ,$$

in such a way that function inside the sum is approximately 1 when the distance R_{IJ} between atom I and J is less than d^0, while it goes to 0 when $R_{IJ} > d^0$; see also Table SI 8. To enhance protonation of ethene, we chose CV1 where we define the coordination number between the ethene carbon atoms and all the hydrogen atoms coordinated to the Rh atoms. CV2 was defined to accelerate the coordination/decoordination of ethene carbon atoms with the Rh atoms. Further, CV3 was chosen for accelerating the decoordination of H atoms that are coordinated to the Rh atoms in the reactant structure.

Table SI 8: CVs employed in the metadynamics simulations of hydrogenation of ethene.

CV label	CV type	d^0 (in Å)
CV1	C^{tot}[C–H]	1.40
CV2	C^{tot}[Rh–C]	2.55
CV3	C^{tot}[Rh–H]	1.90

The free energy surfaces reconstructed from QM/MM and QM/p–MM simulations are given in Figure SI 3. MD simulations were carried out for 12 and 11 ps using QM/MM and QM/p-MM potentials, respectively.

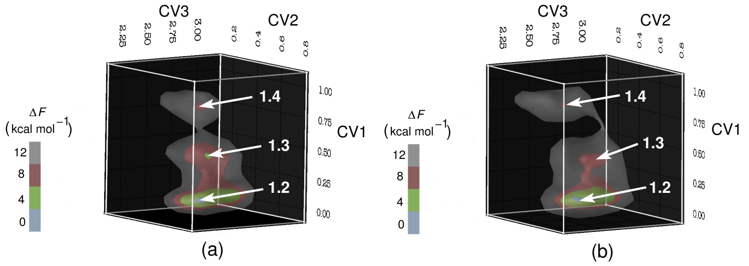

Figure SI 3: The free energy surfaces reconstructed from (a) QM/MM and (b) QM/p–MM metadynamics simulations of hydrogenation of ethene to ethane.

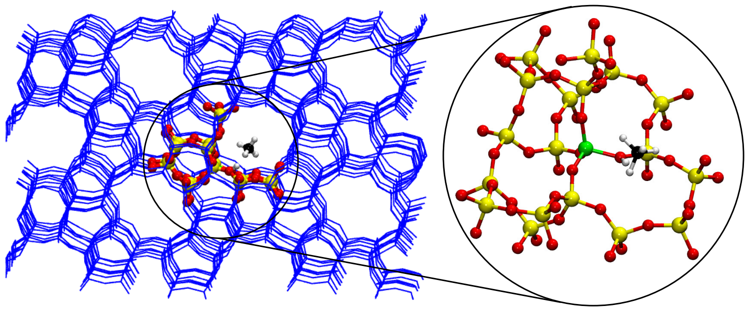

Figure SI 4: QM/MM system used in our simulation of proton exchange between methane and H-ZSM-5 zeolite. Here, the QM atoms are highlighted in CPK format, while the MM atoms are shown in blue sticks. The capping H atoms are not shown here for clarity. Atom colors: Si(yellow), Al(green), O(red), C(black) and H(white).

5 Proton Exchange Reaction Between Methane and H–ZSM–5 Zeolite

In order to model the H–ZSM–5 zeolite, we have taken a supercell of 2×2×2 ($Si_{768}O_{1536}$) in an orthorhombic cell of size 40.341 × 39.498 × 26.527 Å^3. The initial structure was obtained from the zeolite database [2] and then optimized using MZHB [4] and p–MZHB potentials. The optimized lattice parameters were used in these simulations.

The methane molecule and a part (19 tetrahedral sites) of the zeolite structure was treated in the QM region. One of Si atoms in the QM region, which is part of the T12 crystallographic site, is replaced by an Al atom. Negative charge acquired by the zeolite framework due to this substitution is neutralized by a proton added to the lattice oxygen atom adjacent to the substituted Al (see Figure SI 4). The QM region was treated in a tetragonal box of size 20.102 × 20.102 × 18.092 Å^3. Here, the PBE+D2[9, 22] exchange correlation functional was employed. All the other technical details were the same as that in the previous section.

In order to simulate the reaction of interest, we employed the recently proposed TASS (Temperature Assisted Sliced Sampling) technique [25] as implemented in the CPMD program. This technique combines

Table SI 9: List of CVs employed in TASS simulation. d_{I-J} is the distance between atom I and atom J, and, $\phi_{I-J-K-L}$ is the torsional angle between four atoms I, J, K and L. See Figure 7b for atom labels and Figure 7a for the chemical reaction.

CV label	CV type
CV1[a]	$d_{\mathrm{H1-C2}}$ - $d_{\mathrm{C2-H3}}$
CV2	$d_{\mathrm{H3-O8}}$
CV3	$d_{\mathrm{H3-O9}}$
CV4	$d_{\mathrm{H1-O7}}$
CV5	$\phi_{\mathrm{H4-C2-H5-H6}}$

[a]CV sampled in US technique.

the temperature accelerated MD with the biased MD techniques such as umbrella sampling (US) and/or metadyamics, for efficient sampling of high–dimensional free energy surfaces. In this work, we have applied US bias along one of the CVs, while all the CVs were coupled a high temperature (1000 K) bath; see Table SI 9 for the CVs used for enhanced sampling.

The CV temperature was maintained at 1000 K by coupling to a Langevin thermostat, following the extended Lagrangian formalism as in Ref.[25]. Force constant of 0.4 a.u. was used for coupling CVs with their auxiliary degrees of freedom, and a mass of 50.0 a.m.u. was assigned to these auxiliary variables. The umbrella potentials were placed at an interval of 0.2 a.u. along the CV1, and a force constant of 2.0 a.u. was used for all the umbrella potentials. For each umbrella window, we performed MD simulations for 8 ps.

The final free energy surface was reconstructed using the reweighting scheme proposed in Ref.[25], and was projected along CV1 to obtain the Figure 7c in the manuscript.

References

[1] Dollase, W. A. *Z. Kristallogr.*, **1965**, *121*, 369–377.

[2] Zeolite Database, *www.iza-structure.org/databases/.*

[3] Sanders, M. J.; Leslie, M.; Catlow, C. R. A. *J. Chem. Soc., Chem. Commun.*, **1984**, pp 1271–1273.

[4] Sahoo, S. K.; Nair, N. N. *J. Comput. Chem.*, **2015**, *36*, 1562–1567.

[5] Gale, J. D. *J. Chem. Soc., Faraday Trans.*, **1997**, *93*, 629–637.

[6] Hoover, W. G. *Phys. Rev. A*, **1985**, *31*, 1695–1697.

[7] Leach, A. R. *Molecular Modelling: Principle and Applications.* Pearson, Harlow, England, 2nd edition, 2010.

[8] Schlick, T. *Molecular Modeling and Simulation: An Interdisciplinary Guide.* Springer, New York, 2nd edition, 2010.

[9] Perdew, J. P.; Burke, K.; Ernzerhof, M. *Phys. Rev. Lett.*, **1996**, *77*, 3865–3868.

[10] Vanderbilt, D. *Phys. Rev. B*, **1990**, *41*, 7892–7895.

[11] Martyna, G. J.; Klein, M. L.; Tuckerman, M. *J. Chem. Phys.*, **1992**, *97*, 2635–2643.

[12] Nasluzov, V. A.; Ivanova, E. A.; Shor, A. M.; Vayssilov, G. N.; Birkenheuer, U.; Rösch, N. *J. Phys. Chem. B*, **2003**, *107*, 2228–2241.

[13] Schröder, K.-P.; Sauer, J. *J. Phys. Chem.*, **1996**, *100*, 11043–11049.

[14] Hriljac, J.; Eddy, M.; Cheetham, A.; Donohue, J.; Ray, G. *J. Solid State Chem.*, **1993**, *106*, 66 – 72.

[15] Diaz-Cabanas, M.-J.; A. Barrett, P. *Chem. Commun.*, **1998**, pp 1881–1882.

[16] Bibby, D. M.; Dale, M. P. *Nature*, **1985**, *317*, 157–158.

[17] Van Koningsveld, H. *Acta Crystallogr., Sect. B: Struct. Sci.*, **1990**, *46*, 731–735.

[18] Jorgensen, J. D. *J. Appl. Phys.*, **1978**, *49*, 5473–5478.

[19] Downs, R. T.; Palmer, D. C. *Am. Min.*, **1994**, *79*, 9–14.

[20] de Leeuw, N. H.; Parker, S. C. *Phys. Rev. B*, **1998**, *58*, 13901–13908.

[21] Sahoo, S. K.; Nair, N. N. *J. Comput. Chem.*, **2016**, *37*, 1657–1667.

[22] Grimme, S. *J. Comput. Chem.*, **2006**, *27*, 1787–1799.

[23] Laio, A.; Parrinello, M. *Proc. Natl. Acad. Sci.*, **2002**, *99*, 12562–12566.

[24] Iannuzzi, M.; Laio, A.; Parrinello, M. *Phys. Rev. Lett.*, **2003**, *90*, 238302.

[25] Awasthi, S.; Nair, N. N. *J. Chem. Phys.*, **2017**, *146*(9), 094108.
